# Squamous Cell Papilloma of the Urinary Bladder Endoscopically Mimicking Cancer

**DOI:** 10.1155/2013/486312

**Published:** 2013-09-18

**Authors:** Dimosthenis Miliaras, Ioannis Vakalopoulos, Eleftherios Anagnostou

**Affiliations:** ^1^Laboratory of Histology, School of Medicine, Aristotle University, 54006 Thessaloniki, Greece; ^2^Department of Urology, School of Medicine, Aristotle University, 54635 Thessaloniki, Greece; ^3^Department of Histopathology, Euromedica General Clinic, 54645 Thessaloniki, Greece

## Abstract

Squamous cell lesions of the urinary bladder are generally rare. Herein we describe a case of 74-year-old male patient with a benign squamous cell papilloma. Histologically, the tumor presented extensive keratinization at its surface and showed no nuclear atypia or stromal invasion. The tumor cells were negative for HPV DNA. These lesions are extremely rare, and even though they are considered benign and non-HPV related, they should be followed, since recurrence has been reported.

## 1. Introduction

Papillomatous lesions of the urinary bladder are quite common and include both benign and malignant tumors as well as nonneoplastic lesions (metaplastic changes, polyps, and some forms of cystitis). Tumors usually originate from the urothelium of the urinary bladder. When benign, they may be urothelial papillomas or inverted papillomas, and when malignant they may be urothelial carcinomas with or without invasion of the bladder wall. Malignant urothelial tumors outnumber by far their benign counterparts, with the latter being estimated in less than 1% of all bladder tumors [1]. Pure squamous cell carcinoma of the urinary bladder is much more rare than urothelial carcinoma, accounting for only 1.1% and 2.8% of all bladder cancers in men and women, respectively [2]. However, squamous differentiation (intercellular bridges and/or keratinization) may be encountered in 21% of urothelial carcinomas of the bladder [3]. A benign squamous cell papilloma of the urinary bladder appears to be an extremely rare event with only 10 cases reported in the literature [4, 5]. This is a benign proliferative lesion, composed of papillary cores covered by squamous epithelium without koilocytic atypia or dysplasia. Herein we describe a case of this very rare tumor.

## 2. Case Presentation

A 74-year-old male patient admitted to our urology clinic for recurrent episodes of macroscopic hematuria, severe frequency, urgency, and painful voiding during the last six months. His medical history was otherwise unremarkable, except for hypertension. Previous urological investigation had revealed moderate prostate enlargement with normal PSA levels and a digital rectal examination with no signs suggestive of malignancy. The patient was put under tamsulosin and finasteride treatment for benign prostate hyperplasia, and his symptoms were tolerable until the last six months. At this point his blood tests were within normal limits, while his urine samples demonstrated microscopic hematuria without increased inflammatory cells. However, an abdominal CT scan revealed some thickening of the bladder wall. All other organs were within normal limits.

**Figure 1:**
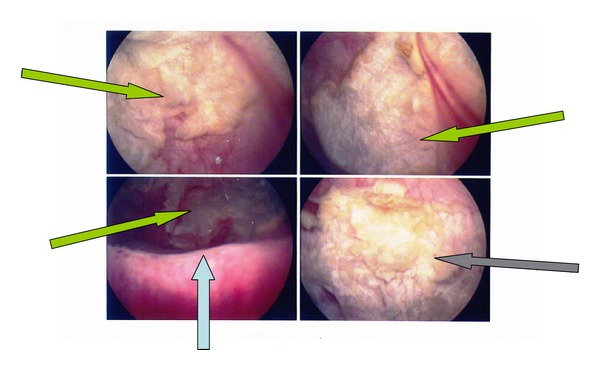
A whitish, slightly exophytic lesion was seen on cystoscopy (arrows).

**Figure 2:**
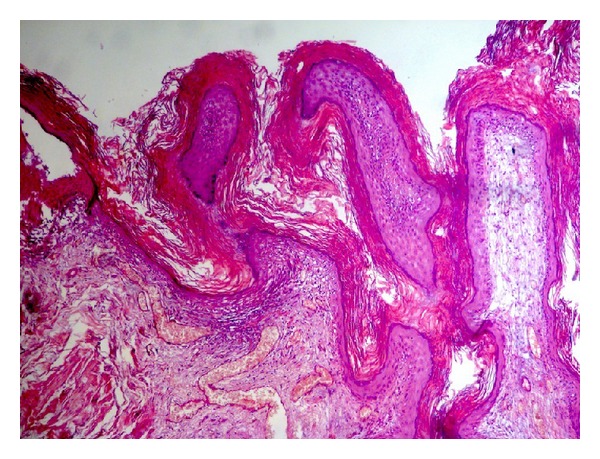
A papillary, exophytic, noninvasive tumor with extensive keratinization on its surface was seen on microscopy (H&E, ×100).

**Figure 3:**
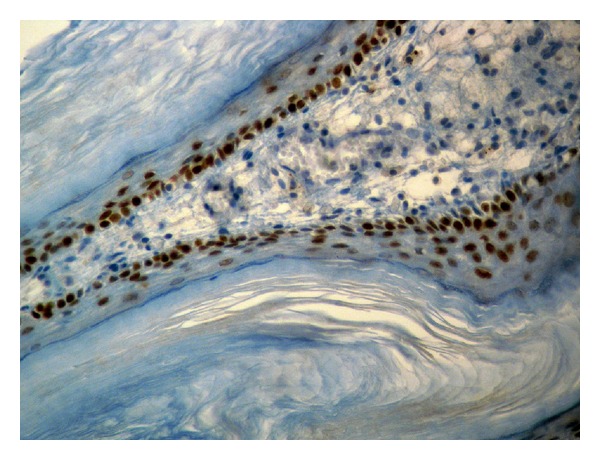
Immunohistochemically, the nuclei of the tumor cells were positive to p63 (DAB/Hematoxylin, ×400).

**Figure 4:**
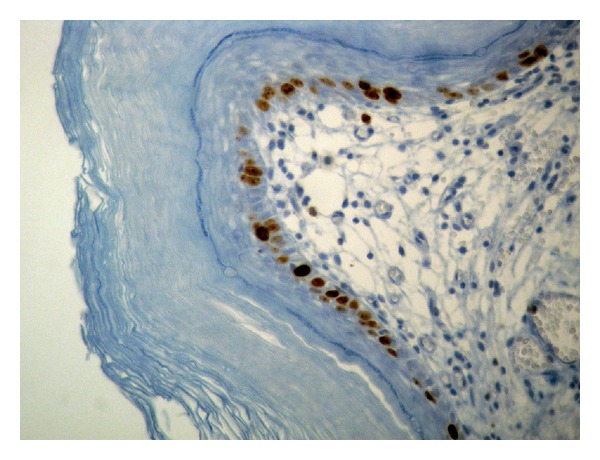
The proliferative activity is limited to the basal layer of the squamous epithelium as highlighted by Ki-67 immunohistochemistry (D.A.B./Hematoxylin, ×400).

On cystoscopy an extensive, whitish, slightly exophytic lesion was found at the mucosa of the floor and the posterior wall of the bladder, measuring 25 × 15 mm. Endoscopically, the lesion was appearing like necrotic tissue and clinically regarded as highly suspicious for an invasive urothelial tumor (Figure 1). A transurethral resection (TUR) of the lesion followed. Histology revealed a benign squamous cell papilloma. It was characterized by multiple papillary fronds, composed of bland, benign appearing squamous cells (Figure 2). There were no morphological features suggestive of human papilloma virus (HPV) infection, and there were no dysplasia, mitoses, or evidence of invasive malignancy. Immunohistochemically, the tumor cells were p63-positive (Figure 3) and cytokeratin 7 and 20 negative. Ki-67 activity was limited to the basal layer of the epithelium (Figure 4). In order to exclude an HPV infection, an HPV DNA detection test was performed. Total DNA was extracted from 10 *μ*m paraffin sections of the tumor, and molecular HPV detection followed using the linear array HPV genotyping test (Roche Molecular Systems, Branchburg, NJ, USA). This proved to be negative for HPV DNA. The postoperational course of the patient was uneventful, and the patient was released from the hospital the next day after the interventional procedure. 

One month after the TUR procedure, the patient's irritative and painful symptoms had been resolved. Cystoscopy and kidney ultrasound scan at three- and six-month postoperative intervals were normal. 

## 3. Discussion

Squamous cell lesions of the urinary bladder are generally rare, and they may be benign or malignant [4–7]. Benign lesions include keratinizing squamous metaplasia, verrucous squamous hyperplasia, squamous cell papilloma, and condyloma acuminatum [4, 5]. Malignant squamous lesions of the urinary bladder include squamous differentiation in urothelial carcinoma, squamous cell carcinoma in situ, and invasive squamous cell carcinoma. Differential diagnosis of a squamous cell papilloma of the urinary bladder should include not only other benign and malignant squamous lesions but also urothelial tumours. Squamous cells tend to be spindle shaped with abundant eosinophilic cytoplasm, usually arranged parallel to the surface, while urothelial cells are mostly columnar with a moderate amount of clear or basophilic cytoplasm, and their long axis lies perpendicular to the surface. In addition, squamous tumors frequently keratinize on their surface when they are exophytic or in the middle of the cell masses when they are invasive. Differential diagnosis may be aided by immunohistochemistry when there is a doubt about the cell of origin, that is, urothelial versus squamous cells. Squamous cells express p63 and high molecular weight keratins, but they are negative to cytokeratin 7 and 20, while urothelial cells usually express both cytokeratin 7 and 20. Distinction of a benign lesion from a malignant lesion will be based on standard histologic criteria, such as nuclear atypia, mitoses, and the degree of stratification and maturation of the epithelium. Proliferation indices like Ki-67 and protein expressions of p53, cyclin D1, or p16 may also be helpful in doubtful cases. Invasion is recognized by the presence of irregular groups of cells inside the stroma. Histologically, our case was benign appearing and noninvasive, and origin from squamous cells was quite evident from the extensive keratinization on the surface of the lesion. In addition, HPV infection was excluded with a molecular method. This is quite important since an HPV-related lesion (condyloma acuminatum) may be related to condylomas in the external genitalia [5] and may recur as an in situ or invasive squamous cell carcinoma [4]. Cases of benign squamous cell papilloma of the urinary bladder should also be followed, since one the ten cases reported in the literature recurred as a low-grade urothelial carcinoma [4].
